# Diving into progress: a review on current therapeutic advancements in spinal muscular atrophy

**DOI:** 10.3389/fneur.2024.1368658

**Published:** 2024-05-24

**Authors:** Pankaj Bagga, Sudhakar Singh, Gobind Ram, Subham Kapil, Avtar Singh

**Affiliations:** ^1^School of Bioengineering & Biosciences, Lovely Professional University (LPU), Phagwara, India; ^2^PG Department of Biotechnology, Layalpur Khalsa College, Jalandhar, India; ^3^Department of Zoology, DAV College Jalandhar, Jalandhar, India; ^4^School of Electrical Engineering and Computing (SoEEC), Adama Science and Technology University (AS-TU), Adama, Ethiopia

**Keywords:** spinal muscular atrophy, survival motor neuron, rehabilitation, nusinersen, onasemnogene abeparvovec, risdiplam

## Abstract

Spinal muscular atrophy (SMA) is an uncommon disorder associated with genes characterized by the gradual weakening and deterioration of muscles, often leading to substantial disability and premature mortality. Over the past decade, remarkable strides have been made in the field of SMA therapeutics, revolutionizing the landscape of patient care. One pivotal advancement is the development of gene-targeted therapies, such as nusinersen, onasemnogene abeparvovec and risdiplam which have demonstrated unprecedented efficacy in slowing disease progression. These therapies aim to address the root cause of SMA by targeting the survival motor neuron (SMN) gene, effectively restoring deficient SMN protein levels. The advent of these innovative approaches has transformed the prognosis for many SMA patients, offering a glimmer of hope where there was once limited therapeutic recourse. Furthermore, the emergence of small molecule compounds and RNA-targeting strategies has expanded the therapeutic arsenal against SMA. These novel interventions exhibit diverse mechanisms of action, including SMN protein stabilization and modulation of RNA splicing, showcasing the multifaceted nature of SMA treatment research. Collective efforts of pharmaceutical industries, research centers, and patient advocacy groups have played an important role in expediting the translation of scientific discoveries into visible clinical benefits. This review not only highlights the remarkable progress achieved in SMA therapeutics but also generates the ray of hope for the ongoing efforts required to enhance accessibility, optimize treatment strategies, rehabilitation (care and therapies) and ultimately pave the way for an improved quality of life for individuals affected by SMA.

## 1 Introduction

Spinal muscular atrophy (SMA) is a rare and debilitating genetic disorder that primarily exerts influence on the motor neurons in the spinal cord, leading to muscle atrophy and weakness. This disorder has garnered significant attention in recent years due to the development of groundbreaking treatments, such as gene therapies, which have the potential to change the prognosis for individuals affected by SMA (1). SMA is a heterogeneous group of inherited neuromuscular disorders characterized by the progressive degeneration of motor neurons in the spinal cord. The condition’s prevalence and incidence can vary significantly based on geographic and ethnic factors, making it a complex subject for epidemiological study (2). The estimated global prevalence of SMA ranges from 1 in 6,000 to one in 10,000 live births, with variations in different populations and regions. This range represents a significant burden for affected families and healthcare systems (3). This condition is an autosomal recessive disorder resulted by mutations in the survival motor neuron 1 (*SMN1*) gene. The incidence of SMA depends on the carrier frequency of these mutations in a given population (4). SMA is classified into different divisions based on the age of onset and clinical severity, which also have implications for epidemiology. SMA type I, with an early outbreak and severe phenotype, is often the most common, while SMA type IV, with adult onset and milder symptoms, is rarer (5). There may be variations in the prevalence and incidence of SMA across different countries and regions. For example, some studies suggest a higher prevalence in certain European populations. Research is ongoing to understand these variations better (6). Addressing the impact of SMA on families and healthcare systems necessitates a multifaceted approach that includes comprehensive support services, caregiver education and training, psychosocial interventions, financial assistance programs, and healthcare system reforms to improve access to specialized care (7, 8). Collaboration among healthcare providers, lawmakers, advocacy organizations, and community stakeholders is critical for reducing the effect of SMA and improving the well-being of afflicted individuals and their families (9). In this review, we discussed the epidemiology and clinical classification of SMA, shedding light on the latest research, rehabilitation and clinical findings.

## 2 Clinical classification of SMA

**Figure 1:**
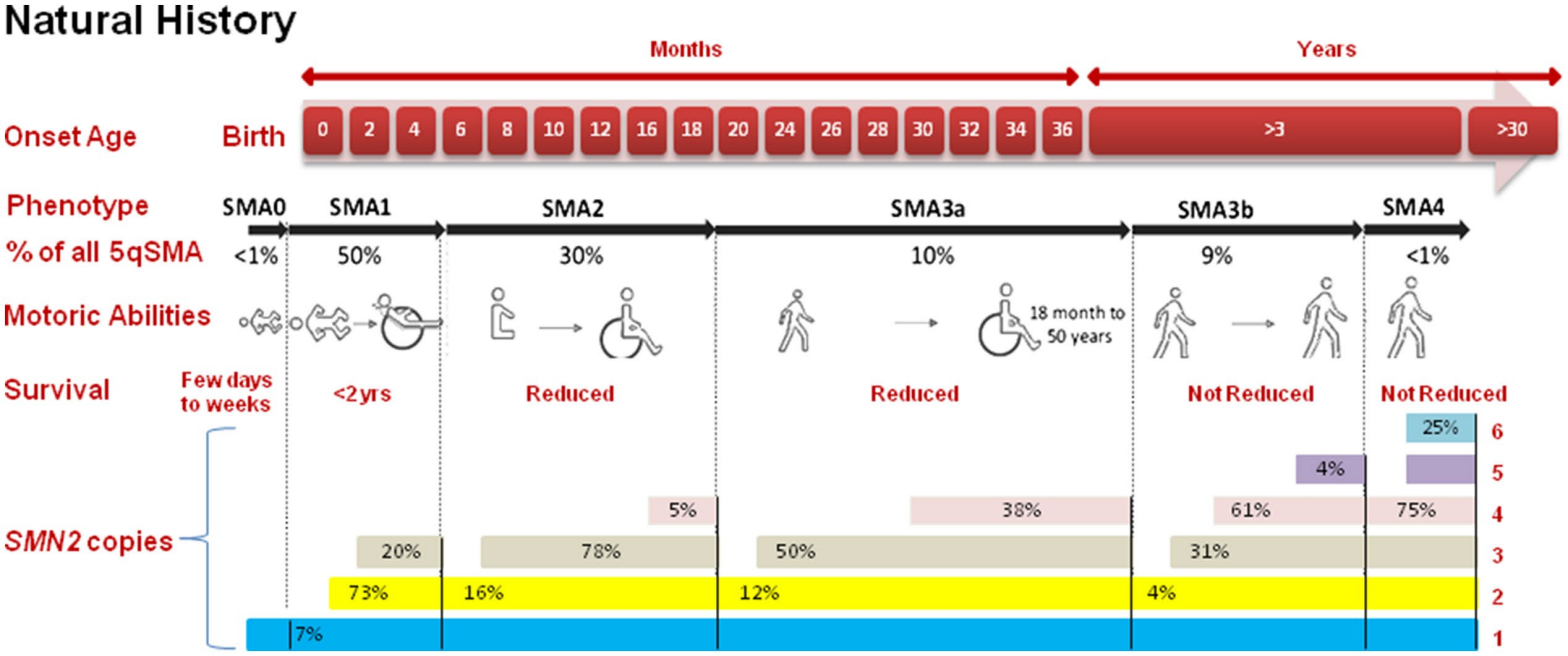
Comprehensive insight into the progression of SMA. The top panel categorizes the five types of SMA (0, 1, 2, 3, 4) which is based on the onset age and achieved motor capabilities. SMA type 3 is further subdivided upto 3a (onset <3 years) and 3b (onset >3 years). Furthermore, the figure presents the total count of *SMN2* gene copies for each SMA type.

In 1891, SMA first came to light through the observations of Guido Werdnig in two infant brothers (10). Over the subsequent nine years, Johann Hoffmann documented an additional seven cases. The traditional framework for classifying SMA was built upon the timing of symptom onset and the highest level of motor function achieved. While the cases they scrutinized had intermediate symptom severity, the term “Werdnig-Hoffmann disease” was coined to denote the more severe manifestations of SMA (11). The year 1899 saw Sylvestre and Beevor describe severe forms of SMA, further delineating the spectrum. In 1964, Dubowitz contributed to the field by detailing intermediate forms of SMA in 12 patients and naming this variant “Dubowitz disease” (11–13). Additionally, in 1955, the discovery of a milder form of SMA was made, with Kugelberg and Welander providing a comprehensive description one year later (14). This division of kinds of SMA is based on the age of symptom when its starts, the highest motor function achieved, and the severity of muscle weakness. Research into the epidemiology of SMA is vital for understanding the wide prevalence of disease, its occurrence, and natural evolution. These studies provide beneficial insights into the biological, ecological, and demographic factors that drive the development and progression of SMA in populations. The International Standard of Care Committee for SMA (ISCCSMA) has classified SMA into five primary types, as shown in the Figure 1, which include:

### 2.1 SMA type 0

This is the most drastic and rarest form of SMA, with onset in womb or within the first few days of life. Neonate with SMA type 0 often exhibit severe muscle weakness and may not survive beyond a few months (15).

### 2.2 SMA type I (Werdnig-Hoffmann disease)

This is the most common form of SMA. Symptoms typically appear before first six months of life, and affected newborn may never will have the ability to sit independently or stand. SMA type I is characterized by severe muscle weakness, respiratory difficulties, and a shortened lifespan if not treated (16).

### 2.3 SMA type II

This form of SMA has a later onset, typically occurring between six and eighteen months of age. Affected individuals often get the ability to sit but may struggle with standing or walking. The rate of disease progression varies among individuals (12).

### 2.4 SMA type III (Kugelberg–Welander disease)

This type of disorder has an onset after 18 months of age. Individuals with this form may achieve the ability to walk independently, but muscle weakness and atrophy progress slowly. Some individuals may experience a relatively normal lifespan (17).

### 2.5 SMA type IV

This is the mildest form of SMA, with an adult onset of symptoms. Affected individuals may experience muscle weakness, twitching, and exercise intolerance. The progression of the disorder is slow, and life aging is normal (18). Twenty cases with SMA type 4 were found in a Brazilian cohort of 227 SMA patients. This study includes the biggest cohort of SMA type 4 patients and provides practical, genetic, radiological, and neurophysiological aspects that may serve as biomarkers for future SMA-specific genetic therapeutics (19).

## 3 Genetics of spinal muscular atrophy

**Figure 2:**
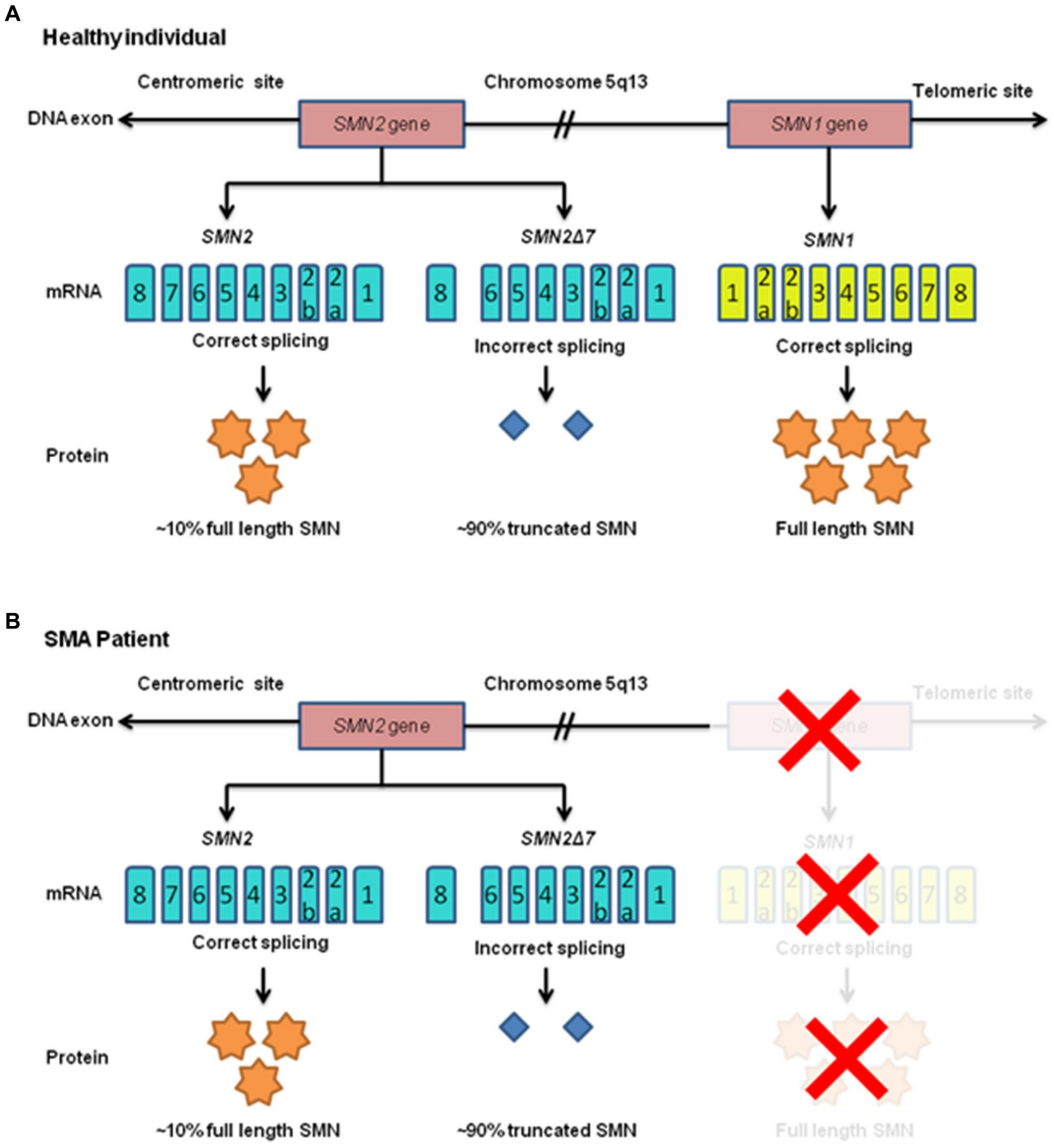
Human survival motor neuron (SMN) gene expression is shown in a schematic diagram for both healthy people and those with SMA. The chromosome 5q13 region (long arm of chromosome 5) has been explicitly identified as the location of the telomeric *SMN1* and centromeric *SMN2* genes. Full-length, functional SMN (FL-SMN) protein is produced by the *SMN1* gene whereas the *SMN2* gene, owing to incorrect splicing, produces 90% truncated SMN protein (SMNΔ7) and only 10% of FL-SMN protein. **(A)** Both SMN genes are present in healthy individuals. **(B)** The *SMN1* gene is absent in SMA patients due to mutations, which prevent *SMN1* from producing FL-SMN protein (this condition is denoted by a red “X”). Because production is completely dependent on the *SMN2* gene, there is inadequate production.

The *SMN1* gene encodes the survival motor neuron (SMN) protein, which is essential for the normal functioning of motor neurons in the spinal cord (20, 21). SMN, a foundational protein within the SMN-Gemin multiprotein complex, serves as a core component. Additionally, it actively engages in various physiological functions, including responding to cellular stress, facilitating axon transport, regulating cytoskeletal dynamics, modulating mitochondrial and bioenergy pathways, and participating in ubiquitin pathways. Consequently, SMN emerges as a significant molecule, intricately involved in a multitude of essential activities that underpin human existence (22). These *SMN* genes are found within the 5q13 region, which harbors inverted repeats and multiple gene copies (20, 23, 24). The telomeric version of *SMN1*, with its nine exons, generates a functional 294-amino acid, 38 kDa SMN protein as shown in Figure 2. Typically, this protein is found in both organalles that is the cytoplasm and nucleus, specifically in the Gemini of coiled bodies compartment, which forms Cajal bodies holding high concentrations of small ribonucleoproteins (snRNPs) along with pre-mRNAs (25). SMN contains crucial and highly conserved domains that are essential for its cellular functions. Any kind of mutations occurring within these domains of *SMN1* result in the production of an inefficient protein (26). The *SMN2* is a centromeric gene which is a paralog of *SMN1*, having almost identical sequences with *SMN1* except for 5 nucleotide differences. To understand the contributions of the survival motor neuron 2 (*SMN2*) gene to spinal muscular atrophy (SMA) pathology, it’s important to grasp its differences in alternative splicing compared to *SMN1* and how these differences impact disease severity and progression. The one of these changes leads to the exclusion of exon number 7 in approximately 90% of the transcripts through alternative splicing (27). *SMN2* is located 875 kb far from *SMN1* and originates from a duplication of an ancestral gene which is unique to the human lineage (28). Both *SMN1* and *SMN2* genes encode the survival motor neuron (SMN) protein, which is crucial for the survival and function of motor neurons. However, a critical difference between *SMN1* and *SMN2* lies in a single nucleotide difference within exon 7, resulting in a C-to-T transition in *SMN2*. This single nucleotide change in *SMN2* affects the alternative splicing pattern, leading to the exclusion (skipping) of exon 7 in a significant proportion of transcripts. Exon 7 skipping results in the production of an isoform of the SMN protein lacking exon 7 (SMNΔ7), which is less stable and less functional compared to the full-length SMN protein produced by *SMN1*. The exclusion of exon 7 in a substantial portion of *SMN2* transcripts results in reduced levels of functional SMN protein in cells, contributing to the pathogenesis of SMA. While *SMN2* can partially compensate for the loss of *SMN1* function, the lower levels of full-length SMN protein produced by *SMN2* are insufficient to fully support motor neuron survival and function. The severity and progression of SMA are influenced by the number of copies of *SMN2* present in the genome. Individuals with fewer copies of *SMN2* typically produce lower levels of functional SMN protein and tend to have more severe forms of the disease, whereas those with more copies of *SMN2* may produce higher levels of functional SMN protein and exhibit milder symptoms. The unique alternative splicing pattern of *SMN2* has made it a primary target for therapeutic interventions aimed at increasing the production of full-length SMN protein. SMA symptoms manifest when there is a deficiency of proper functional SMN protein, usually stemming from minimum one copy of the *SMN1* (29). However, around 10% of full-length *SMN2* transcripts, often present in multiple copies within the genome, provide some degree of protection against motor neuron degeneration (30). The more *SMN2* copies a patient possesses, the more they can compensate for the absence of *SMN1* (31). Consequently, in rare cases, individuals with 6 or more copies can exhibit milder symptoms appearing after the age of 30, characterized by mild muscle weakness and retained full mobility. Most type I SMA patient’s carries either one or two *SMN2* copies (32). While the number of *SMN2* gene copies strongly correlates with disease severity, some studies suggest that it may not always be a definitive indicator of severity, especially in SMA patients who retain one *SMN1* allele (33). Additionally, even when *SMN* is expressed normally, point mutations in *SMN* can affect protein functionality and stability, leads to the disorder, along with genetic and epigenetic factors, as well as environmental influences, may modulate disease (34). Approaches such as antisense oligonucleotide (ASO) therapy and small molecule drugs target the splicing machinery to promote the inclusion of exon 7 in *SMN2* transcripts, thereby increasing the production of functional SMN protein. These therapies aim to augment the levels of functional SMN protein in motor neurons, potentially ameliorating disease symptoms and improving outcomes for individuals with SMA.

## 4 Molecular mechanisms of SMA

The underlying molecular mechanisms of SMA revolve around the loss of functional SMN protein and its impact on motor neurons and muscle cells. The reduction in functional SMN protein in motor neurons results in their degeneration (35). Motor neurons are responsible for transmitting signals from the spinal cord to muscles, and without proper functioning SMN protein, these neurons become vulnerable to damage and eventual death. This leads to muscle weakness and atrophy (36). SMN protein plays a crucial role in the assembly of snRNPs, which are essential for mRNA splicing (37). Impaired snRNP assembly due to SMN deficiency leads to widespread splicing defects in various genes, further exacerbating motor neuron dysfunction (38). While SMA is primarily a disorder of motor neurons, the resulting muscle atrophy and weakness are critical clinical features. The lack of neural input from affected motor neurons causes muscle disuse, contributing to muscle wasting (39). The neuromuscular junction, where motor neurons communicate with muscle cells, is also affected in SMA (40). The loss of functional motor neurons disrupts this communication, leading to muscle weakness and contractures (41).

## 5 Diagnostic approaches of spinal muscular atrophy

SMA presents with a spectrum of clinical features, ranging from severe, early-onset forms to milder, adult-onset forms. The key clinical manifestations include muscle weakness, atrophy, and hypotonia. Patients with SMA may also exhibit respiratory difficulties, joint contractures, and scoliosis (42). It is essential to recognize these clinical signs early, as timely intervention can significantly impact the prognosis and quality of life for affected individuals.

### 5.1 Clinical evaluation

A thorough clinical evaluation is the initial step in diagnosing SMA. The healthcare provider takes a detailed medical history, conducts a physical examination, and assesses motor function (43). The clinical evaluation includes a review of family history to identify any known cases of SMA or related neuromuscular disorders. It is important to consider that SMA may not be the first suspicion in cases with mild or atypical symptoms (44).

### 5.2 Electromyography

Electromyography (EMG) is a diagnostic technique used to assess the electrical activity of muscles and nerves (45). In SMA, EMG may reveal neurogenic changes, denoting motor neuron dysfunction. EMG can help distinguish SMA from other neuromuscular disorders and provide information about the extent of motor neuron involvement (46).

### 5.3 Nerve conduction studies

Nerve conduction studies (NCS) evaluate the function of peripheral nerves. In SMA, NCS can be normal or show mild abnormalities. These tests help rule out other neurological conditions and provide additional information to support the diagnosis of SMA (47).

### 5.4 Muscle biopsy

While muscle biopsy is not the primary diagnostic tool for SMA, it can be used to confirm the absence of muscle pathology, ruling out conditions like muscular dystrophy. Muscle biopsies typically show atrophy and denervation in SMA, supporting the diagnosis (48).

### 5.5 Serum creatine kinase levels

Measuring serum creatine kinase (CK) levels can be useful in differentiating SMA from muscular dystrophies. In SMA, CK levels are usually within the normal range or only mildly elevated, whereas in muscular dystrophies, CK levels are significantly elevated (49).

### 5.6 Newborn screening

Newborn screening for spinal muscular atrophy (SMA) is a critical and rapidly evolving aspect of pediatric healthcare aimed at early detection and intervention for this devastating genetic disorder. One of the primary benefits of newborn screening for SMA is the early identification of affected infants. Unlike in the past, when diagnosis often occurred after the onset of symptoms, newborn screening allows for early intervention and treatment (50).

### 5.7 Genetic testing

Genetic testing is the gold standard for diagnosing SMA. It provides a definitive diagnosis, identifies the specific genetic mutation, and helps determine the severity of the condition. Genetic testing typically involves the following approaches: (a) the primary genetic test for SMA involves analyzing the *SMN1* gene. Most SMA cases result from deletions or mutations in this gene, leading to reduced SMN protein levels. This test is highly specific and sensitive and can diagnose SMA with a high degree of accuracy (51). (b) In addition to *SMN1* analysis, counting the number of copies of the *SMN2* gene can provide information about the disease severity. SMA patients with more *SMN2* copies tend to have milder forms of the disease, while those with fewer copies typically have more severe forms (52).

### 5.8 Next-generation sequencing

Next-generation sequencing (NGS) is a powerful tool for identifying rare or typical mutations in the *SMN1* gene. It can be especially useful in cases where standard genetic tests do not yield a diagnosis. NGS can also detect other rare genetic conditions that may mimic SMA (53).

### 5.9 Prenatal testing

Genetic testing can be performed during pregnancy to identify SMA in the fetus. This can be done through chorionic villus sampling or amniocentesis. Early diagnosis allows for informed reproductive decisions and early intervention if the fetus is affected (54). Prenatal testing for spinal muscular atrophy (SMA) raises several ethical considerations, including issues related to informed consent, autonomy, disability rights, and the potential for discrimination. It’s crucial that expectant parents fully understand the purpose, benefits, limitations, and potential consequences of SMA prenatal testing. They should have access to comprehensive information about SMA, including its prognosis, available treatments, and the emotional impact of receiving a positive result. Current recommendations by the American College of Medical Genetics (ACMG) include offering SMA carrier screening to all couples, regardless of race or ethnicity, before conception or early in pregnancy. Current recommendation by the American Congress of Obstetricians and Gynecologists (ACOG) do not advise preconception and prenatal screening for SMA be offered to the general population and advice testing offered to general population (55). Some disability rights advocates argue that prenatal testing for conditions like SMA perpetuates ableism and sends a message that individuals with disabilities have less value or are not worthy of existence. This perspective challenges the notion that certain disabilities should be actively prevented or eliminated through selective abortion.

## 6 Disease-modifying treatments and current implications

There are several treatments approaches for the SMA such as (Table 1).

**Table 1:** Treatment options currently in use for spinal muscular atrophy.

Treatment	Nusinersen (Spinraza)	Onasemnogen abeparvovec (Zolgensma)	Risdiplam (Evrysdi)
Class	Antisense oligonucleotide	Adeno-associated virus (AAV) based gene therapy	Small molecule
Mechanism	Improves *SMN2* splicing to produce full-length SMN protein	Provides a functioning SMN trans gene	Improves *SMN2* splicing to produce full-length SMN protein
Administrative route	Intrathecal injection	Intravenous injection	Oral
FDA approved age categories	All	Greater than 2 years	More than two months
Frequency	Dosing schedule: 4 loading doses in the first 2 months, then every 4 months	Just one time dose (single dose)	Daily
Problems with current treatments	Unable to get a lumbar puncture done	AAV9 antibodies present at the baseline	Interactions between drugs
FDA approval	December 2016	May 2019	August 2020
Cost	$125,000 per dosage (approx.)	$2.125 million per treatment	$100,000–$340,000 annually
Unfavourable outcomes	Lumbar puncture problems, proteinuria, Thrombocytopenia	Transaminitis, thrombocytopenia, troponemia, and acute liver damage	Diarrhoea, rash, and fever

Nusinersen (marketed as Spinraza^®^) was the first FDA-approved disease-modifying treatment for SMA having obtained approval in December 2016 and by the EMA in 2017 for both infant and adult (56). This innovative therapeutic approach, which involves the intrathecal administration of a 2′-O-methoxyethyl phosphorothioate modified antisense oligonucleotide (ASO), focuses on enhancing the incorporation of exon 7 into mRNA transcripts of *SMN2* (57). The intrathecal route of administration is crucial for nusinersen’s effectiveness in treating SMA because it allows for targeted delivery to the site of pathology, bypasses the blood-brain barrier, optimizes concentration at the target site, minimizes systemic side effects, and provides a longer duration of action within the central nervous system (57). By administering nusinersen directly into the central nervous system (CNS) through the intrathecal route, the medication effectively suppresses the activity of certain splice-factors and binds to a specific intronic splice-silencing site within intron 7 of *SMN2* (58). This intervention substantially increases the probability of exon 7 being included in the mRNA, ultimately enabling the translation of a more substantial quantity of fully functional SMN protein (27). This enhanced production has demonstrated significant improvements in both survival and the overall condition of various experimental models of SMA. Importantly, nusinersen’s journey to approval and commercialization has been bolstered by a multitude of studies confirming its efficacy, without any notable drug-related adverse events (59). Due to ASOs’ inability to traverse the blood-brain barrier, nusinersen was consistently administered intrathecally in all clinical trials. During the initial loading phase, it was administered four times over two months, and in the maintenance phase, it was given once every four months (60). The standard dosage of nusinersen typically amounts to 12 milligrams (61). Studies on nusinersen have revealed the potential for some patients to regain lost abilities, such as sitting up, standing, and walking, without the need for therapy. Furthermore, early initiation of this treatment has demonstrated positive outcomes in individuals with SMA types I, II, and III (62). It is worth noting that a notable drawback of this therapy is the possibility of side effects, including constipation and upper and lower respiratory tract infections (63).

Onasemnogene abeparvovec (Zolgensma), an advanced gene therapy: In May 2019, the FDA granted approval to AVXS-101, also known as Zolgensma is a gene therapy approved for the treatment of SMA, developed by AveXis, a subsidiary of Novartis (56). This approval followed the release of favorable outcomes from the phase one clinical trial known as START (Identifier: NCT01547871). This trial assessed the drug’s safety and effectiveness when administered as a one-time infusion to infants with SMA symptoms appearing before six months of age. Subsequently, in March 2020, Zolgensma received conditional marketing authorization, and in May 2020, it was granted approval by the European Medicines Agency (EMA) as well (Zolgensma, 2020a; Novartis, 2020) (64). The adeno-associated virus 9 (AAV9) capsid is used to transport the SMN-encoding complementary DNA (cDNA) to the motor neurons that need it (65–67). A single dose of AVV9 administered intravenously (IV) is sufficient to transport a functional copy of the *SMN1* gene over the blood-brain barrier and into patient cells, where it may stimulate the production of SMN protein (27). The *SMN1* trans gene and synthetic promoter based on AVV9 are also crucial components in maintaining SMN protein synthesis throughout time (68). Although it successfully corrects the underlying molecular defect in SMA, it has a deleterious effect on the liver by elevating serum amino transferase (69). However, prednisone is effective at reducing elevated liver enzymes. Therefore, at least three months after administration, patients should be monitored for liver function (70, 71). The long-term durability of the benefits of Zolgensma (onasemnogene abeparvovec) is still being actively studied and monitored. Zolgensma is a gene therapy approved for the treatment of spinal muscular atrophy (SMA) in pediatric patients, and it has shown remarkable efficacy in improving motor function and survival in clinical trials. While the initial data from clinical trials and real-world experience have shown sustained benefits of Zolgensma treatment over several years, including improvements in motor function and survival, more long-term follow-up is needed to fully understand the duration of these benefits. Clinical trials and observational studies are ongoing to assess the durability of Zolgensma’s effects, including its impact on motor function, respiratory function, quality of life, and survival rates over extended periods. These studies involve monitoring patients treated with Zolgensma for several years to track their progress and detect any potential changes in treatment outcomes over time. It’s important to note that as research continues and more data become available, our understanding of the long-term benefits and potential limitations of Zolgensma treatment will continue to evolve. Patients and caregivers should work closely with healthcare providers to stay informed about the latest research findings and recommendations regarding the use of Zolgensma in the management of SMA (65, 72).

Risdiplam (Evrysdi^™^) was authorized by the FDA as of 7 August 2020, as the first oral medication for children as young as 2 months old and adults with SMA. It is a collaborative development effort involving Roche, PTC Therapeutics Inc., and the SMA Foundation, aimed at addressing spinal muscular atrophy (56, 73). Risdiplam serves as a modifier of mRNA splicing that leads to an elevation in SMN protein expression (74). It is a tiny molecule that changes the splicing of the *SMN2* by binding to two locations in the *SMN2* pre-mRNA. These sites are known as the 5′ splice site (5′ ss) of intron number 7 and the exon splicing enhancer 2 (ESE2) of exon 7 (75). Increases in full-length SMN mRNA and protein levels are caused by the unique specificity of binding two sites, which also reduces impact on other pre-mRNA splicing and prevents the likelihood of off-target effects (76). According to preclinical studies, risdiplam can reach the central nervous system and peripheral organs *in vivo* and can result in a significant increase of SMN protein in the blood, brain, and muscles, as well as an increase in survival in various SMA mouse models (77). While risdiplam’s systemic distribution in preclinical tests with oral administration allowed for the possibility of an impact on other tissues, nusinersen’s intrathecal delivery method mostly limited its effect to motoneurons of the central nervous system (78). Previous studies in human and murine models suggest that SMA may in fact be considered a multi-system disorder involving the neuromuscular junction, cardio-vascular system, lung, gastrointestinal-tract, and liver (79). Risdiplam has shown significant improvements in motor function and has the advantage of being an oral therapy, making it a more convenient option for many patients (80).

SMA treatments have evolved notably in recent years, particularly as previously stated gene therapies like Zolgensma and disease-modifying drugs like Spinraza (nusinersen). Much more significant perspective, which should consider insurance coverage, availability across regions, and efforts to improve access to SMA treatments. While these drugs offer a promising treatment option for children with SMA, they are highly costly, as seen in Table 1. According to studies, certain governments have made efforts to design policies for such disease treatments, such as: The Department of Revenue, Ministry of Finance, govt. of had issued Notification No. 46/2021-Customs dated 30.09.2021, which waives all Basic Customs Duty (BCD) and Integrated Goods and Services Tax (IGST) on drugs imported (personal use only) for the treatment of spinal muscular atrophy (SMA) rare disease, making medicines for SMA rare disease more affordable (81). Government of India also made a provision for financial assistance of up to Rs. 50 lakhs to patients suffering from any category of Rare Diseases such as SMA and for treatment at any of the Centres of Excellence (CoE) identified in the NPRD-2021, outside of the Rashtriya Arogaya Nidhi umbrella scheme (82). The National Health Service (NHS) England, for example, states that Biogen (the pharmaceutical company that manufactures treatment for SMA) will make the treatment for spinal muscular atrophy (SMA) available to the youngest and most severely affected (SMA type 1) patients immediately, with NHS England offering funding contingent on the National Institute for Health and Care Excellence (NICE) publication of final guidance. In Singapore, the Rare Disease pay has been established to pay five drugs to treat three rare diseases. In Malaysia and Australia, qualifying patients are given discounted access to pricey and life-saving medications (83).

## 7 Rehabilitation (care and therapies) and disease management for spinal muscular atrophy

**Figure 3:**
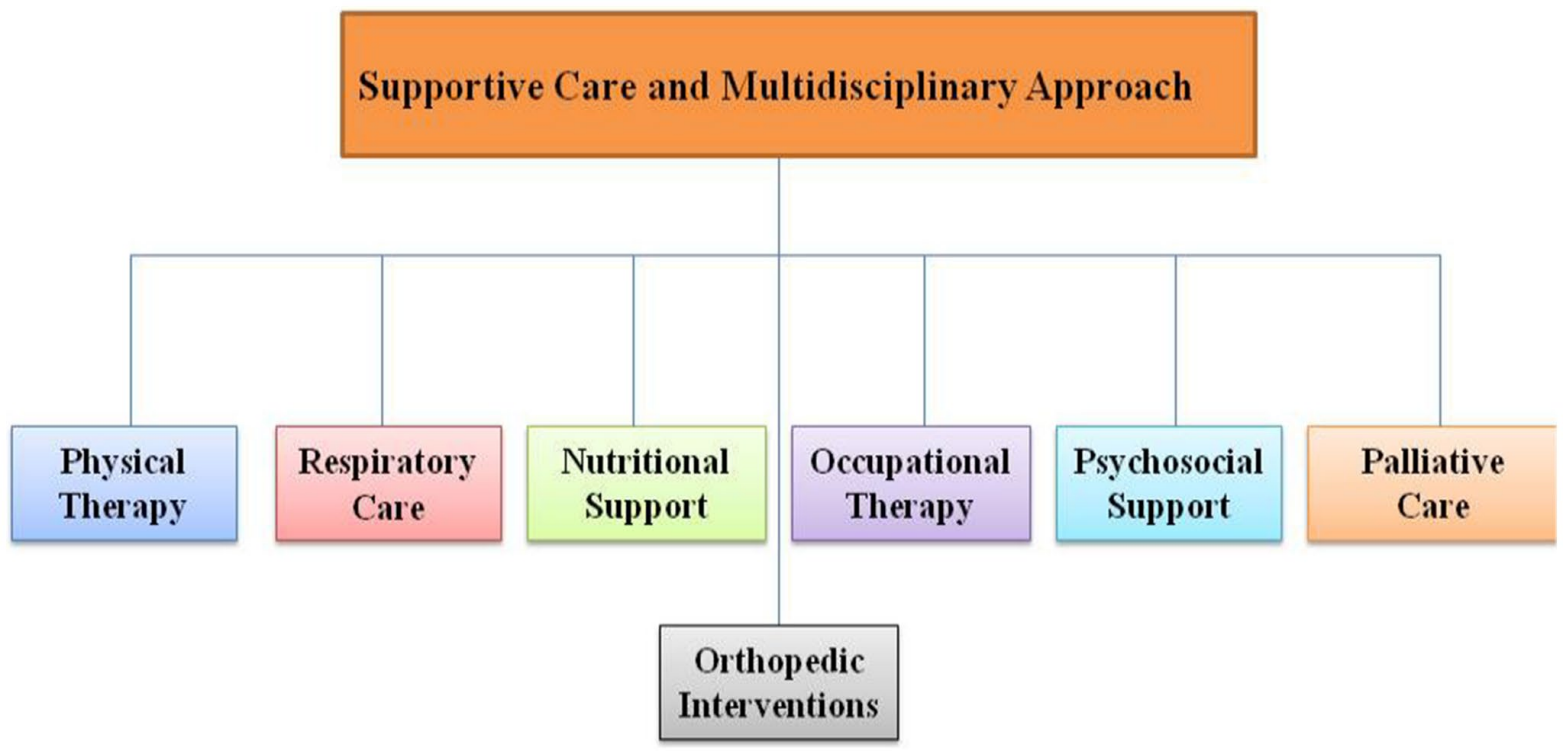
Supportive care and multidisciplinary approach flow chart.

In addition to disease-modifying treatments, SMA management often involves a multidisciplinary approach as shown in Figure 3 that focuses on addressing the symptoms and complications associated with the disease. Supportive care strategies aim to improve the quality of life for individuals with SMA and include:

### 7.1 Physical therapy

Physical therapy is essential in managing muscle weakness, contractures, and maintaining range of motion (84, 85). People affected by spinal muscular atrophy may experience limitations in their ability to fully articulate their joints due to muscle weakness. This can potentially lead to the development of contractures, characterized by muscle tightness that may become permanent and restrict mobility (86). Physical therapy often incorporates exercises and stretching routines designed to enhance flexibility and overall functionality (87). These interventions aim to minimize the likelihood of joint contractures, mitigate the progression of scoliosis, and promote the maintenance of a healthy weight. Mounting evidence suggests that consistent participation in physical therapy sessions can yield positive outcomes in terms of both function and the progression of spinal muscular atrophy in patients (86, 87).

### 7.2 Respiratory care

The management of the respiratory system plays a pivotal role in addressing spinal muscular atrophy (88). Weakness in the chest muscles can hinder one’s capacity to breathe or cough effectively, consequently elevating the likelihood of infections (89, 90). The available respiratory care solutions encompass both non-invasive and invasive methods: (i) non-invasive respiratory care comprise strategies that aim to circumvent or postpone the necessity for invasive procedures (88). Specialized apparatus, such as a ventilator or a bilevel positive airway pressure (BiPAP) machine, can provide a constant airflow to the lungs using a mask that covers the mouth and/or nose. Additionally, a home-based cough assist device may be utilized to facilitate coughing and the clearance of secretions. (ii) Invasive respiratory care establishes a protected passage to the lungs using an endotracheal tube inserted either through the mouth (intubation) or directly into the trachea via a minor neck incision (tracheotomy) (91, 92).

### 7.3 Nutritional support

The weakening of muscles can result in some individuals with SMA experiencing a decline in their ability to chew and swallow proficiently. This situation increases the risk of food or liquids being aspirated into the lungs, potentially leading to respiratory infections (93, 94). To address this, a temporary or permanent feeding tube may be inserted to ensure the essential intake of nutrition and hydration (93). Broadly speaking, there are two categories of feeding tubes: those inserted through the nose and those placed in the abdominal area. Nasogastric (NG) tubes are inserted through the nasal passage and deliver nutrition directly into the stomach (95, 96). These are typically employed for patients requiring short-term feeding tube access and are conveniently replaceable (95). Gastrostomy (G) tubes are surgically implanted through the abdominal wall and directly into the stomach (97–99). Due to their ease of maintenance, they are often the favored choice for individuals in need of extended-term feeding assistance.

### 7.4 Orthopedic interventions

Orthopedic surgery may be required in cases where there are severe joint contractures or scoliosis (98, 100). Scoliosis, characterized by a spinal curvature, can be a challenge for individuals with spinal muscular atrophy as a consequence of muscle weakness (101). An orthopedic specialist may propose postural support in the form of bracing or recommend surgical intervention to address scoliosis (98, 101).

### 7.5 Occupational therapy

Occupational therapists work with SMA patients to enhance their ability to perform daily tasks and improve their independence (102, 103). They assess patients’ needs and recommend assistive devices or home modifications to facilitate daily activities. Their role includes: adaptive techniques means teaching individuals adaptive techniques and recommending assistive devices that enable greater independence (104). Home modifications means assessing home environments and suggesting modifications to make daily tasks more manageable (105). Assisting with communication means in cases of severe SMA, occupational therapists can help individuals use communication devices to facilitate interaction with others (106).

### 7.6 Psychosocial support

SMA affects not only the physical health but also the emotional and psychosocial well-being of patients and their families (107). Ensuring the holistic care of a patient with SMA and their family is insufficient without the monitoring and treatment of their psychosocial welfare. Requirements differ based on the patient’s age and specific sub-types of SMA. Psychosocial health can be influenced by various elements, including social and emotional factors as well as treatment factors such as innovative therapies. Psychosocial care should encompass a wide range of characteristics, including social and cognitive development, quality of life, and the impact on patient and family functioning in various situations such as home, school, or job. The care of SMA should include the involvement of a mental health practitioner, such as a psychologist, psychiatrist, or neuropsychologist, as well as a social worker who has specialised experience in assisting patients with chronic diseases. Assessments should be considered around the time of diagnosis, before entering school, and after a change in functionality. Implementing psychologically informed care and employing a range of interventions has the potential to mitigate psychological morbidity in both children and parents. During every multidisciplinary appointment, it is important to assess the individual’s mental health and quality of life. If deemed essential, the mental health clinician will be involved in evaluating the psychological state of the patient, as well as their parents and siblings (107). Psychosocial support addresses these aspects and may include: (i) counseling: individual and family counseling can help individuals and caregivers cope with the emotional challenges of living with SMA (107, 108). (ii) Support groups: joining support groups, either in person or online, can provide a sense of community and shared experiences. (iii) Mental health services: access to mental health services is essential for addressing anxiety, depression, and stress that may result from the condition (108, 109).

### 7.7 Palliative care

For individuals with advanced SMA or those with severe complications, palliative care can provide symptom management, pain relief, and emotional support to enhance quality of life (110).

### 7.8 Disease management approaches for SMA management

#### 7.8.1 Supportive care and multidisciplinary approach

The management of SMA requires a multidisciplinary team approach involving various healthcare professionals to address the diverse needs of individuals with the condition. The team may include neurologists, physical and occupational therapists, respiratory therapists, nutritionists, orthopedic surgeons, and social workers. This collaborative approach ensures that the physical, emotional, and psychosocial aspects of SMA are managed comprehensively (111). Supportive care is the fundamental aspect of clinical management in spinal muscular atrophy (SMA). The development of disease-modifying medications such as nusinersen, onasemnogene abeparvovecxioi, and risdiplam has provided improved treatment choices for the most severe forms of the condition. These medications have increased survival rates and brought hope for a longer and better quality of life. Additionally, they have influenced the way healthcare is provided for these patients. Although there have been some improvements in the field, adults living with SMA and those transitioning into adulthood have been somewhat overlooked, despite the emergence of studies and advancements such as enhanced respiratory care, home adaptations, and devices that promote greater independence, like power wheelchairs and voice amplifiers. It is important for everyone to acknowledge and appreciate these achievements. The effects of fragmented care might be intensified for individuals migrating from paediatric to adult care, as they no longer receive the same degree of coordinated assistance provided in paediatric settings. According to reports, individuals who are transferring from paediatric to adult healthcare services face challenges in understanding and interacting with a complex health system and new specialists. They commonly describe this experience as “challenging and intimidating.”

In addition, adults with SMA may have difficulties during this phase of transition, particularly when they need to relocate (e.g., for higher education), as this necessitates them to become part of a new healthcare system. Although not prevalent within the SMA community as a whole, certain jurisdictions have acknowledged this issue and have adopted targeted measures to mitigate its effects. For instance, they have established transitional clinics where medical professionals from both adult and paediatric fields participate in appointments. We advocate for increased implementation of optimal methods and specialised procedures (such as established transitional care clinics, communication paths between paediatric and adult experts, and a nationwide network of specialists) that streamline the transition to adult care and the transfer of knowledge (46).

#### 7.8.2 Assistive devices and technology

The use of assistive devices and technology plays a significant role in SMA management. These devices help individuals with SMA lead more independent and fulfilling lives (112). Examples include: wheelchairs, scooters, and other mobility aids provide individuals with the freedom to move and explore their environments (113). Augmentative and alternative communication (AAC) devices assist those with severe SMA in expressing themselves (106). These systems enable individuals to control various aspects of their environment, such as lights, doors, and appliances, through adapted technology. Adapting the home environment with features like ramps, wider doorways, and accessible bathrooms enhances accessibility (105). Adaptive technology and software allow individuals with limited physical mobility to use computers and access the internet (104).

## 8 Emerging therapies

Research into SMA continues, with several promising therapies under investigation. These therapies include small molecule drugs, gene-editing technologies, and exon-skipping therapies, among others. The goal is to further enhance the disease-modifying potential and offer a more comprehensive treatment approach for SMA. Advances in genetic testing have made it easier to diagnose SMA accurately and offer prenatal testing for at-risk pregnancies. These advancements include: NGS has become a powerful tool in identifying rare or atypical mutations in the *SMN1* gene and other related genes. It can uncover genetic variations that were previously challenging to detect (114, 115). Techniques like chorionic villus sampling and amniocentesis allow for the diagnosis of SMA in the fetus, enabling informed reproductive decisions and early intervention if the fetus is affected (116, 117).

### 8.1 Gene-editing technologies

Emerging gene-editing techniques, such as CRISPR-Cas9, offer the potential to correct genetic mutations directly, providing a curative approach to SMA. These technologies are in the early stages of development and are being explored in preclinical studies. In addition to this, CRISPR technology, which expands the scope of genetic engineering and gene treatments, enables the treatment of a wide range of hereditary illnesses. Some prior research in the literature show that SMA can be treated using the CRISPR method. Homology directed repair (HDR)-based CRISPR technology, which produces a high rate of in-del (insertion-deletion) mutations rather than editing, has been proven unsuitable for therapeutic purposes. CRISPR-prime editing (PE) technology is a novel type of gene editing technique that enables precise genomic alterations without the need for double-strand breaks or donor DNA sequences. The CRISPR-prime editing approach has also been employed in rare disorders like as sickle cell anaemia and Tay–Sachs, and its effectiveness in editing diverse harmful variants has been proven. However, CRISPR Prime Editing-mediated gene editing for spinal muscular atrophy (SMA) has yet to be investigated (114).

### 8.2 Small molecule therapies

Small molecules that target specific pathways involved in SMA are also under investigation. These drugs aim to increase SMN protein production and improve motor function (116).

### 8.3 Combination therapies

Researchers are exploring the use of combination therapies, including a mix of *SMN2*-targeting drugs and other treatments, to enhance the efficacy of SMA management (118). Combining different therapeutic strategies to maximise SMA treatment outcomes is an exciting approach. Limited data supports the efficacy of expensive drug combinations in people, encouraging clinicians and scientists to examine all therapeutic options (56, 119) A combination of SMN-dependent ASO-inducing *SMN2* exon inclusion and SMN-independent myostatin inhibition yielded positive results in a SMA animal model (56, 120). A limited sample of patients were treated with a combination of Zolgensma and nusinersen, but the long-term benefits remain unclear. Zolgensma and nusinersen have distinct modes of action, making drug-to-drug interactions less common. Nusinersen targets an intron sequence to increase exon 7 inclusion. The transplanted Zolgensma gene lacks introns and hence should not interfere with nusinersen translation. Zolgensma treatment should be approached with caution due to the reported adverse event of thrombocytopenia associated with nusinersen. Long-term follow-up data, particularly in pre-symptomatic patients, is needed to evaluate the effectiveness and hazards of combination therapy (56). Combination therapies, treatments and advocacy initiatives have played an important role in determining research orientations and legislative changes in the management of spinal muscular atrophy (SMA). Advocacy groups such as cure SMA, SMA foundation, and fight SMA have been instrumental in catalyzing SMA research. By raising awareness, funding research initiatives, and fostering collaborations among scientists, these organizations have accelerated the pace of discovery in understanding the underlying genetic mechanisms of SMA, identifying potential therapeutic targets, and developing innovative treatment strategies.

### 8.4 Early intervention and pre-symptomatic treatment

Research has shown the benefits of early intervention, even before the onset of symptoms, in infants with SMA (121). The nurture study demonstrated that early treatment with nusinersen in pre-symptomatic infants significantly improved motor function and developmental outcomes (122).

### 8.5 Patient and caregiver advocacy

SMA patient and caregiver advocacy groups have played a crucial role in raising awareness, driving research, and improving access to care and treatments. Their efforts have been instrumental in advancing the SMA field (123).

## 9 Conclusion

Thirty years after the discovery of the SMN gene, global scientific efforts have successfully transformed spinal muscular atrophy (SMA) into a manageable condition. Three distinct drugs utilizing cutting-edge technology—gene therapy, antisense oligonucleotides (ASOs), and small molecules focused on *SMN2* splice correction—have gained approval from both the FDA and EMA, demonstrating remarkable enhancements, particularly when administered prior to the onset of symptoms. SMA, known for its substantial economic impact, has become even more financially demanding with the introduction of these novel therapies. Addressing the considerable economic burden associated with these treatments has led to an increasing advocacy for the inclusion of SMA in newborn screening (NBS) programs. While the existing therapies are likely sufficient for immediate administration after birth in cases of intermediate and mild SMA, questions arise regarding whether individuals with only two *SMN2* copies will follow a trajectory similar to their age-matched counterparts and if they may require additional SMN-independent therapies. Comprehensive longitudinal studies are imperative to explore potential new phenotypes linked to these innovative therapies. Despite the presence of biomarkers associated with disease progression, further investigations are needed to identify potential non responders, enabling them to transition to alternative therapies. SMA treatments are often expensive, prompting concerns regarding equal access for all patients, especially in low-income areas or nations with limited healthcare resources. Addressing pricing and guaranteeing access to these life-changing medications for all SMA patients worldwide remains a serious concern. Numerous unanswered questions persist, necessitating meticulous future research. Nonetheless, SMA stands as a model illustrating how genetic insights can pave the way for the development of targeted therapies.

## Author contributions

PB: Formal analysis, Data curation, Writing – original draft. SS: Writing – review & editing, Supervision, Writing – original draft. GR: Writing – review & editing, Supervision. SK: Writing – original draft, Visualization. AS: Writing – review & editing, Supervision, Formal analysis.
